# An anticancer Os(II) bathophenanthroline complex as a human breast cancer stem cell-selective, mammosphere potent agent that kills cells by necroptosis

**DOI:** 10.1038/s41598-019-49774-x

**Published:** 2019-09-16

**Authors:** Vojtech Novohradsky, Lenka Markova, Hana Kostrhunova, Zdeněk Trávníček, Viktor Brabec, Jana Kasparkova

**Affiliations:** 1 Czech Academy of Sciences, Institute of Biophysics, Kralovopolska 135, 612 65 Brno, Czech Republic; 2 Division of Biologically Active Complexes and Molecular Magnets, Regional Centre of Advanced Technologies and Materials, Faculty of Science, Palacky University, Slechtitelu 27, 783 71 Olomouc, Czech Republic; 3 Department of Biophysics, Faculty of Science, Palacky University, Slechtitelu 27, 783 71 Olomouc, Czech Republic

**Keywords:** Mechanism of action, Cancer stem cells

## Abstract

Conventional chemotherapy is mostly effective in the treatment of rapidly-dividing differentiated tumor cells but has limited application toward eliminating cancer stem cell (CSC) population. The presence of a very small number of CSCs may contribute to the development of therapeutic resistance, metastases, and relapse. Thus, treatment failure by developing novel anticancer drugs capable of effective targeting of CSCs is at present a major challenge for research focused on chemotherapy of cancer. Here, we show that Os(II) complex **2** [Os(*η*^6^-*p*cym)(bphen)(dca)]PF_6_ (*p*cym = *p*-cymene, bphen = bathophenanthroline, and dca = dichloroacetate), is capable of efficient and selective killing CSCs in heterogeneous populations of human breast cancer cells MCF-7 and SKBR-3. Notably, its remarkable submicromolar potency to kill CSCs is considerably higher than that of its Ru analog, [Ru(*η*^6^-*p*cym)(bphen)(dca)]PF_6_ (complex **1)** and salinomycin, one of the most selective CSC-targeting compounds hitherto identified. Furthermore, Os(II) complex **2** reduces the formation, size, and viability of three-dimensional mammospheres which more closely reflect the tumor microenvironment than cells in traditional two-dimensional cultures. The antiproliferation studies and propidium iodide staining using flow cytometry suggest that Os(II) complex **2** induces human breast cancer stem cell death predominantly by necroptosis, a programmed form of necrosis. The results of this study demonstrate the promise of Os(II) complex **2** in treating human breast tumors. They also represent the foundation for further preclinical and clinical studies and applications of Os(II) complex **2** to comply with the emergent need for human breast CSCs-specific chemotherapeutics capable to treat chemotherapy-resistant and relapsed human breast tumors.

## Introduction

Many malignancies include a small population of cancer stem cells (CSCs) that self-renew and produce other more differentiated (non-stem) cells which ultimately form the bulk of the tumor-cell population. The CSCs give rise also to metastases and can also act as a reservoir of cancer cells that may cause relapse after surgery, radiation or chemotherapy. The origin of CSCs is unclear, although their formation depends on both genetic and epigenetic alterations^1,2^.

The conventional therapies do not kill the CSCs so that the tumor soon grows back (often with enhanced resistance to the previously used therapy and of a more aggressive nature). Therefore, effective cancer treatments must attack both rapidly-dividing differentiated (non-stem) cancer cells and CSCs including disruption of the microenvironments supporting these cells. By targeting CSCs, it would be possible to treat patients with aggressive, non-resectable tumors, as well as preventing patients from metastasizing and relapsing. Hence, CSCs are important targets for the development of new anticancer therapies^3,4^ so that currently the development of new compounds which kill all cancer cells and in this way broaden a limited spectrum of anticancer agents, including CSCs is a hot area in medicinal chemistry and pharmacology^5,6^.

It is now generally recognized that metal-containing agents may offer the unique therapeutic potential to treat cancer as well. A number of platinum and other transition metal-based compounds have been shown to exhibit antitumor activity in tumor cells, but their efficacy to attack CSCs have not been mostly tested. Interestingly, none of the clinically used metal-based anticancer agents, such as platinum cytostatics, showed potency against CSCs at their therapeutically administered doses. On the other hand, the anti-CSC properties of some metal-based compounds have already been demonstrated (reviewed in ref.^6^). Interestingly, the mechanism of antiproliferative action of the anti-CSC metallodrugs so far successfully tested is distinctly different from that of conventional platinum(II) antitumor drugs^7,8^.

**Figure 1:**
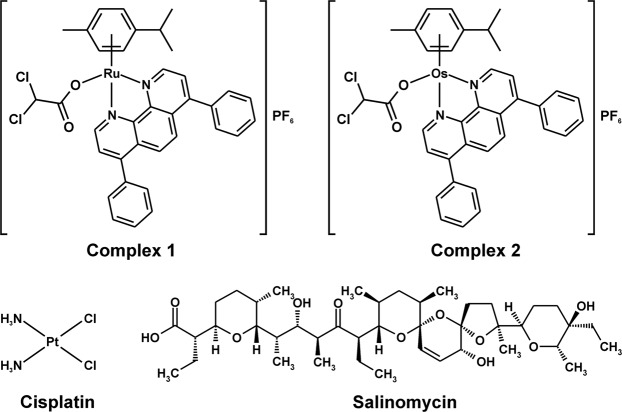
Structural formulae of cisplatin and breast cancer stem cell-selective compounds investigated in this work.

Recently, new half sandwich Ru(II) and Os(II) bathophenanthroline complexes [Ru(*η*^6^-*p*cym)(bphen)(dca)]PF_6_ (Ru(II) complex **1**) and [Os(*η*^6^-*p*cym)(bphen)(dca)]PF_6_ (Os(II) complex **2**) [*p*cym = 1-methyl-4-(propan-2-yl)benzene (*p*-cymene), bphen = 4,7-diphenyl-1,10-phenanthroline (bathophenanthroline); dca = dichloroacetate] have been prepared and characterized^9^ (Fig. 1). As they have shown superior activity in very aggressive triple negative breast cancer cells MDA-MB-231^10^, we decided to investigate the *in vitro* efficacy of these half sandwich Ru(II) and Os(II) complexes using human breast CSCs (hBCSCs). The data reveal that in particular, the Os(II) complex **2** is more effective against hBCSCs than conventional salinomycin, a well-established CSC-potent agent known to have CSC-selective potency^11–13^ as described in this report. In addition, necroptosis rather than apoptosis or autophagy appears the predominant mode of death of hBCSCs.

## Results and Discussion

### Inhibition of mammosphere formation

The search for new anti-CSC compounds has been impeded by limited accessibility and sustainability of CSC-rich cell cultures^14^. Therefore, we first decided to prepare, sort and characterize the cancer cell populations enriched with CSCs (for details, see the Experimental section). Breast cancer MDA-MB-231 cells, in which Ru(II) and Os(II) complexes **1** and **2** (Fig. 1) have shown excellent antiproliferative activity^10^, are unable to form long-term mammosphere cultures (forming rather amorphous aggregates instead of compact spheroids in serum free spheroid suspension culture)^15^. Therefore, next we used hBCSC enriched human breast cancer cell lines (MCF-7^CD44+/CD24−^ and SKBR-3^CD44+/CD24−^) as more convincing CSC models to assess the CSC specificity of the Ru(II) and Os(II) complexes **1** and **2**. For comparative purposes cisplatin (FDA-approved platinum(II) anticancer drug known to have no CSC-selective potency) and salinomycin (compound known to have CSC-selective potency) were used in these experiments as well (Table 1). CD44^+^/CD24^−^ phenotype is commonly used as a reliable phenotype for isolation and characterization of hBCSCs^16^. hBCSCs overexpress CD44^17^, a cell-surface glycoprotein associated with invasion, migration, adhesion, cell proliferation and angiogenesis. Conversely to high expressions of CD44 in MCF-7^CD44+/CD24−^and SKBR-3^CD44+/CD24−^ cells, low expressions of CD24 contribute to the increased ability of cancer cells to grow and metastasize^18^. Thus, our strategy was to isolate CSC population from MCF-7 and SKBR-3 based on the expression of cell surface markers CD44 and CD24 and perform antiproliferative activity tests on mammospheres formed from these MCF-7^CD44+/CD24−^ and SKBR-3^CD44+/CD24−^ suspensions of CSC-enriched single cells (*vide infra*). The results obtained with these CSC-enriched cells were compared with those obtained with original (unenriched) MCF-7 and SKBR-3 cells.

**Table 1:** IC_50_ values (mean values, µM]^a^ for the investigated compounds in human mammospheres determined using CellTiter-Glo^®^ 3D Cell Viability Assay.

IC_50_ (μM)^a^	Cisplatin	Salinomycin	1	2	dca
MCF-7	23 ± 3	7 ± 1	2.8 ± 0.2	2.6 ± 0.4	>100
MCF-7^CD44+/CD24−^	31 ± 2	5.3 ± 0.9	1.3 ± 0.1	0.58 ± 0.07	>100
CSC selectivity index (MCF)^b^	0.7	1.3	2.2	4.5	
SKBR-3	6 ± 1	1.2 ± 0.3	1.5 ± 0.3	1.0 ± 0.2	>100
SKBR-3^CD44+/CD24−^	8.0 ± 0.7	0.8 ± 0.1	0.71 ± 0.09	0.32 ± 0.09	>100
CSC selectivity index (SKBR)^b^	0.8	1.5	2.1	3.1	

^a^IC_50_ values were determined by using CellTiter-Glo^®^ 3D cell viability assay on 144-h old spheroids treated with the investigated compounds for 72 h; results are expressed as mean values ± SD of three independent experiments. ^b^CSC selectivity index was calculated as IC_50_ of unsorted MCF-7 or SKBR-3 cells/IC_50_ of enriched MCF-7^CD44+/CD24−^ or SKBR-3^CD44+/CD24−^ cells.

MCF-7 and SKBR-3 cells exhibit a stable CSC-like population of 2.5 and 5.7%, respectively (Supplementary Figs S1 and S2). These MCF-7^CD44+/CD24−^and SKBR-3^CD44+/CD24^ cells were sorted out for subsequent studies. Cells sorted using fluorescence-activated cell sorting (FACS) were used immediately for further experiments and the status of CD44^+^/CD24^−^ population was checked.

In this study, we first investigated the ability of Ru(II) and Os(II) complexes **1** and **2** and for comparative purposes also cisplatin and salinomycin to inhibit the growth of the mammospheres preformed from MCF-7, SKBR-3, MCF-7^CD44+/CD24−^, and SKBR-3^CD44+/CD24−^ single-cell suspensions. Cells growing in complex 3D cultures more closely reflect the tumor microenvironment than cells in traditional 2D cultures. Hence, 3D culturing cells are much more representative of the *in vivo* environment than 2D cultures. Moreover, hBCSCs tend to form de novo tumor-like structures called mammospheres in non-adherent, serum-free cell cultures^8,19^ so that the capacity of an investigated agent to reduce proliferation of the cells in 3D culture provides a reliable marker for CSC potency^15^.

The antiproliferative effects (the IC_50_ values, the concentration required to reduce viability by 50%) on 3D hBCSC mammospheres were ascertained using CellTiter-Glo^®^ 3D Cell Viability Assay^20^ as described in the Experimental section. The IC_50_ values were derived from dose–response curves (Supplementary Figs S3 and S4) and are summarized in Table 1.

Os(II) complex **2** displayed remarkable submicromolar potency to reduce the number of viable cells in the mammospheres from CSC-enriched MCF-7^CD44+/CD24−^ cells, which was considerably higher than that of Ru(II) complex **1** (2.2-fold), salinomycin (9.1-fold), and cisplatin (53.4-fold). The potency of Os(II) complex **2** was considerably higher (4.5-fold) than that from unsorted MCF-7 cells. In this respect, the effect of Ru(II) complex **1** was similar although less pronounced. Notably, both Ru and Os(II) complexes **1** and **2** were more selective to reduce the number of viable cells in the mammospheres from CSC-enriched MCF-7^CD44+/CD24−^ cells than salinomycin. The effects of the investigated compounds on mammospheres from SKBR-3 and SKBR-3^CD44+/CD24−^ were qualitatively similar. Unsurprisingly, cisplatin showed only very low toxicity in the mammospheres from the investigated human breast cancer cell lines and effectivity rather towards mammospheres from unsorted MCF-7 and SKBR-3 cells. This finding is consistent with the very low effectiveness of cisplatin to reduce the number of viable cells in the mammosphere from hBCSC single-cell suspensions confirming the inability of this anticancer platinum drug to obliterate the entire population of tumor cells, including CSCs.

**Figure 2:**
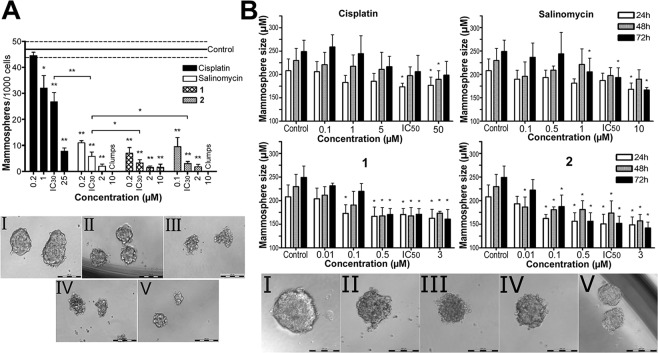
(**A**) Upper panel: Quantification of the formation of the mammospheres made from the suspension of MCF-7^CD44+/CD24−^ single cells untreated and treated with the investigated compounds. The cells were treated with increasing concentrations of the investigated compounds and equitoxic concentrations corresponding to their respective IC_30,72h_ values (determined using CellTiter-Glo^®^ 3D Cell Viability Assay after 72 h of treatment, cisplatin 10.2 μM, salinomycin 0.63 μM, **1** 0.52 μM, **2** 0.15 μM). Cells were incubated for 5 days, and mammospheres with a diameter above 40 μm were counted. Results are expressed as the mean number of mammospheres formed from 1000 viable single cells. Dashed lines represent the range of the SD determined for the control (untreated) cells. Error bars indicate SDs from three independent experiments each made in octuplicate. Cell clumps were not included in the analysis. Statistical analysis was calculated with non-parametric students´ *t*-test; the symbols (*) and (**) denotes a significant difference (*p* < 0.05) from the untreated control. Bottom panel: Representative bright-field images of the mammospheres formed from MCF-7^CD44+/CD24−^ untreated or after 5 days of the treatment with salinomycin and complexes **1** and **2** at their respective IC_30,72h_ (see the legend to the upper panel of this Fig. 2A). I, Untreated control; II, cisplatin; III salinomycin; IV, **1**; V, **2**. Scale bars represent 200 μm. (**B**) Growth inhibitory effects of the investigated compounds in the preformed MCF-7^CD44+/CD24−^ mammospheres. The preformed mammospheres (the mean diameter was~200 μm) were treated with the increasing concentrations (in the range of 0.1–50 µM as indicated in Fig. 2A, upper panel) of the investigated compounds and also with their equitoxic concentrations corresponding to IC_50_ values determined for the treatment lasting 72 h shown in Table 1 (columns marked in Fig. 2A, upper panel as IC_50_) for 72 h, visualized every 24 h of the treatment and analyzed for their sphere mass. Upper panel: Quantification of the mammosphere formation. Bottom panel: Representative bright-field images of the mammospheres obtained after 72 h of the treatment at their IC_50_ values. I, control; II, cisplatin; III, salinomycin, IV, complex **1**; V, complex **2**. Cells were seeded in quadruplicate. Scale bars represent 200 μm. Error bars indicate SDs from three independent experiments each made in octuplicate. Statistical analysis was calculated with non-parametric students´ *t*-test; the symbol (*) denotes a significant difference (*p* < 0.05) from the untreated control.

We also assessed the ability of Ru and Os(II) complexes **1** and **2** and for comparative purposes cisplatin and salinomycin to inhibit the formation of mammospheres from the suspension of MCF-7^CD44+/CD24−^ single cells using the mammosphere formation assay (Fig. 2A). An impetus to this study was given by the observation that the tendency of hBCSC to form mammospheres is connected with the unlimited self-renewal ability of CSCs in non-adherent, serum-free cultures^8,19^. The investigated compounds were added to the suspension of MCF-7^CD44+/CD24−^ single cells at their non-lethal concentrations corresponding to IC_30_ (determined using CellTiter-Glo® 3D Cell Viability Assay after 72 h of treatment; cisplatin 10.2 μM, salinomycin 0.63 μM, **1** 0.52 μM, **2** 0.15 μM) or to their equimolar concentrations ranging from 0.2 μM to 25 μM; the cells were incubated for additional 5 days and analyzed using tumor formation assay.

Addition of salinomycin and Ru(II) and Os(II) complexes **1** and **2**, significantly reduced the number of mammospheres formed relative to the untreated control (upper panel of the Fig. 2A). Complexes **1** and **2** reduced mammosphere formation to a similar extent as salinomycin. In contrast, cisplatin reduced mammosphere formation markedly less effectively. Microscopy studies (bottom panel of the Fig. 2A) revealed that incubation with salinomycin or complexes **1** and **2** resulted in the mammospheres the size of which was also considerably smaller; by contrast, the size of the mammosphere upon addition of cisplatin was smaller only moderately. Moreover, the treatment with equimolar concentrations (10 μM, corresponding to IC_30_ for cisplatin) of salinomycin and Os(II) complex **2** resulted to the complete growth inhibition and loss of the sphere morphology (led to loosely adhered clumps of cells in suspension culture that were not considered proper mammospheres^21^) (upper panel of Fig. 2A). This finding indicated a potent inhibitory effect of Os(II) complex **2** comparable or even better than that of salinomycin on MCF-7^CD44+/CD24−^ mammosphere formation.

To further quantify the inhibitory effects of the investigated compounds on hBCSC mammospheres, the activity towards preformed spheroids was also tested by monitoring the spheroid diameter changes. The 96 h-old spheroids of MCF-7^CD44+/CD24−^ cells with the mean diameter of ~200 μm were treated with the increasing concentrations (in the range of 0.1–50 µM) of the investigated compounds and also with their equitoxic concentrations corresponding to IC_50_ values shown in Table 1 (columns marked in Fig. 2B, upper panel as IC_50_); the spheroids were continuously analyzed every 24 h for the next 3 days to determine their mean diameter using a phase-contrast microscope (Fig. 2B, bottom panel).

The control (untreated) preformed mammospheres showed a continual increase of solid mass characterized by the growth of sphere diameter by approximately 28 μm every 24 h. No significant difference of solid mass characterized by the sphere diameter found for control mammospheres was observed if the mammospheres were treated with cisplatin even at its highest concentration used for the treatment (50 μM) and the longest treatment time (72 h). A small but significant decrease of the solid mass relative to control was found for the mammospheres treated with salinomycin and Ru(II) complex **1**. Notably, Os(II) complex **2** showed a more pronounced inhibitory effect on the preformed mammospheres.

The results shown in Table 1 and Fig. 2 show that Ru and Os(II) complexes **1** and **2** markedly reduced the number of viable cells in the mammospheres from CSC-enriched bCSCs and size of these preformed mammospheres. Also notably, **1** and **2** inhibited the formation of mammospheres from the suspension of MCF-7^CD44+/CD24−^ single cells relative to the untreated control. The effects of complexes **1** and **2** were comparable or even slightly more pronounced than those of salinomycin (an established CSC-potent agent) and Os(II) complex **2** was more effective than Ru(II) complex **1**. On the other hand, cisplatin affected the bCSCs mammospheres considerably less confirming the low effectiveness of cisplatin to affect hBCSCs. Moreover, the treatment with equimolar concentrations (10 μM, corresponding to IC_30_ for cisplatin) of salinomycin and Os(II) complex **2** resulted in the complete growth inhibition and loss of the sphere morphology (led to loosely adhered clumps of cells in suspension culture that were not considered proper mammospheres^21^) (upper panel of Fig. 2A). This observation indicated a potent inhibitory effect of Os(II) complex **2** comparable or even better than that of salinomycin on MCF-7^CD44+/CD24−^ mammosphere formation. Collectively, these findings strongly support the view that in particular Os(II) complex **2** is capable of inhibiting hBCSC self-renewal.

### The effect on the heterogeneity of breast cancer cells

**Figure 3:**
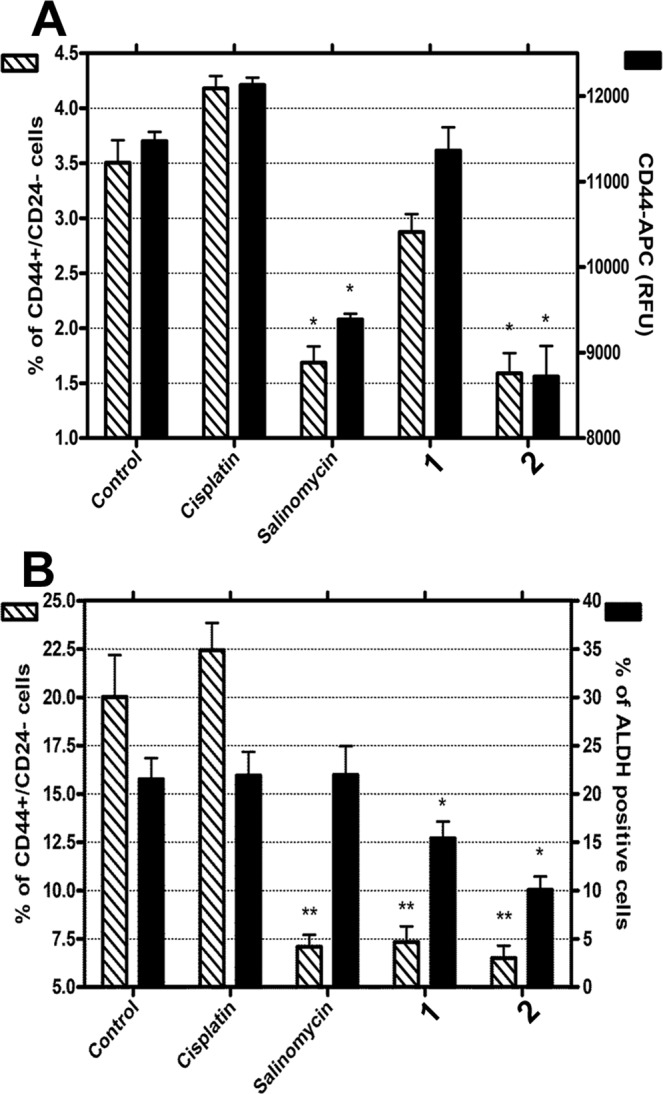
(**A**) The effect of the investigated compounds on the proportion of CSC-like, CD44+/CD24− cells within a heterogeneous population of MCF-7 cells. The cells were treated for 24 h with equitoxic concentrations of the investigated compounds corresponding to their respective IC_50,72h_ (determined by CellTiter-Glo^®^ 3D cell viability assay). Data are expressed as the percentage of CD44+/CD24− cells from the total population (hatched bars) or analyzed for the mean fluorescence intensities of CD44-APC (black bars). Error bars indicate the SDs, statistical analysis was calculated with ANOVA with subsequent non-parametric students´ t-test; the symbol (*) denotes a significant difference (*p* < 0.05) from the sham-treated control. Data are the mean ± SD obtained from at least three independent experiments. (**B**) Evaluation of the inhibitory effects of the investigated compounds on SKBR-3^CD44+/CD24−^ cells. Population analysis; percentage evaluation of population shifts based on CD44/CD24 cell surface markers and ALDH functional marker. Cells were treated with the equitoxic concentrations of the investigated compounds corresponding to their respective IC_50,72h_ values (Table 1). Data are expressed as the percentage of the parental population. Stars at the top of the significant bars were calculated by using ANOVA with subsequent students´ *t*-test and denote significant difference from control (untreated) sample with *p < 0.05 or **p < 0.001.

Next, we investigated the effect of the tested compounds on the population heterogeneity of breast cancer cells MCF-7 and SKBR-3. First, the experiments were performed with MCF-7 cells used as the primary culture, cultivated for at least 5 days under sphere-forming conditions, but without previous sorting by FACS. By monitoring the change in the CD44+/CD24− cell population upon treatment with a given compound, its CSC specificity can be determined^22^. Thus, flow cytometric studies were performed to evaluate the effect of the investigated compounds on the proportion of CSC-like, CD44+/CD24− cells within a heterogeneous population of MCF-7 cells. In these studies, expression of cell surface markers CD44 stained with allophycocyanin (APC) and CD24 stained with phycoerythrin (PE) was assayed (Figs 3A, S5 and S6).

The results of flow cytometric studies (Supplementary Figs S5 and S6) were analyzed for the proportion of CSC-like, CD44+/CD24− cells and for relative fluorescence yielded by APC stained anti-CD44 antibody (Fig. 3A). Upon incubation (24 h) of MCF-7 cells with cisplatin at the concentration corresponding to its respective IC_50,72 h_, the fraction of CD44+/CD24− cells population slightly increased (Fig. 3A, hatched column for cisplatin), suggestive of CSC enrichment and the tendency of cisplatin to induce CSC enrichment rather than CSC depletion^8^. Ru(II) complex **1** at the equitoxic concentration only slightly decreased the percentage of CD44+/CD24− population implying only low efficiency of **1** to induce CSC depletion. In contrast, a marked decrease of the fraction of CD44+/CD24− subpopulation was observed when MCF-7 cells were treated with equitoxic concentrations of salinomycin or Os compound **2** (Fig. 3A, hatched columns for salinomycin and complex **2**). Further, relative fluorescence intensity emitted by anti-CD44 APC antibody-stained MCF-7 cells reflected the overall population shifts observed in MCF-7 cells (Fig. 3A, black columns). These results are consistent with the hypothesis and support the view that in particular Os(II) complex **2** preferentially reduces the proportion of CSC-like, CD44+/CD24− cells within a heterogeneous population of MCF-7 cells. Contrastingly, cisplatin preferentially reduces the proportion of differentiated (non-stem) cancer cells.

A different approach was used to assess the effect of the investigated compounds on the proportion of CSC-like cells within a heterogeneous population of SKBR-3 cells. In this case, we used CSC-enriched SKBR-3^CD44+/CD24−^ cells (*vide supra*) to obtain more pronounced effects of the investigated compounds on the proportion of CSC-like, CD44+/CD24− cells within a heterogeneous population of hBCSC. Importantly, contrary to MCF-7 cells, SKBR-3 cells are ALDH positive (ALDH = aldehyde dehydrogenase), and thus, ALDH activity can serve as another reliable marker for CSC characterization for SKBR-3 cells^23^. Moreover, a functional marker ALDH was found to be associated with CSC characteristics^24^ and has been proposed for use also as the marker for isolation of hBCSC^25^. It has also shown that ALDH positive hBCSC cells are highly tumorigenic, with differentiation and renewal properties^24^. Therefore, fluorescence properties of ALDH activity indicator [BODIPY-aminoacate (BAA)] (Supplementary Figs S7 and S8) combined with antibodies for CD44/CD24 cell surface markers stained with APC and PE (Supplementary Figs S9 and S10) were used in flow-cytometric studies to evaluate the effect of the investigated compounds on the proportion of CSC-like cells within a heterogeneous population of CSC enriched SKBR-3^CD44+/CD24−^ cells.

SKBR-3^CD44+/CD24−^ cells represent a stable, approximately 20% CSC-like cells which displayed ~16% of ALDH-positive cells (Fig. 3B). These cells were treated with the investigated compounds for 24 h at the concentrations corresponding to their respective IC_50,72h_ values and assayed first for the expression of CD44/CD24 cell surface markers (Fig. 3B, hatched columns). The treatment with cisplatin resulted in an additional ~2% enrichment of CD^44+^/CD^24−^ population. A significant diminution of the number of CSC-like CD^44+^/CD^24−^ cells was observed after the treatment of the SKBR-3^CD44+/CD24−^ cells with the equitoxic concentrations of salinomycin, Ru compound **1** and Os compound **2**, reaching only 6–7% of that found in the untreated control from the parental population. We also monitored the distribution of SKBR-3^CD44+/CD24−^ cells in the samples treated with the investigated compounds by detecting cells that expressed ALDH (Fig. 3B, black columns). The amount of SKBR-3^CD44+/CD24−^ cells containing the ALDH marker qualitatively correlated with SKBR-3^CD44+/CD24−^ cells containing CD44/CD24 cell surface markers if the cells were treated with cisplatin and complexes **1** or **2** (Fig. 3B, hatched columns). The experiments aimed at clarifying the reasons why the population of SKBR-3^CD44+^/^CD24−^ cells determined by monitoring ALDH activity in these cells was not affected by salinomycin are in progress in our laboratory and will be published in a separate communication. Nevertheless, the results based on monitoring ALDH activity in SKBR-3^CD44+/CD24−^ cells indicate that the treatment with Ru(II) complex **1** reduced the proportion of SKBR-3^CD44+/CD24−^ cells to a lesser extent than Os(II) complex **2**. Moreover, these results also provide further evidence that Os(II) complex **2** selectively kills bCSC-like cells over bulk cancer cells.

Collectively, the results of the experiments investigating the effect of the tested compounds on the population heterogeneity of breast cancer cells MCF-7 and SKBR-3 (Fig. 3) highlight the remarkable potency and selectivity of Os(II) complex **2** to kill hBCSCs.

### Mode of cell death

**Figure 4:**
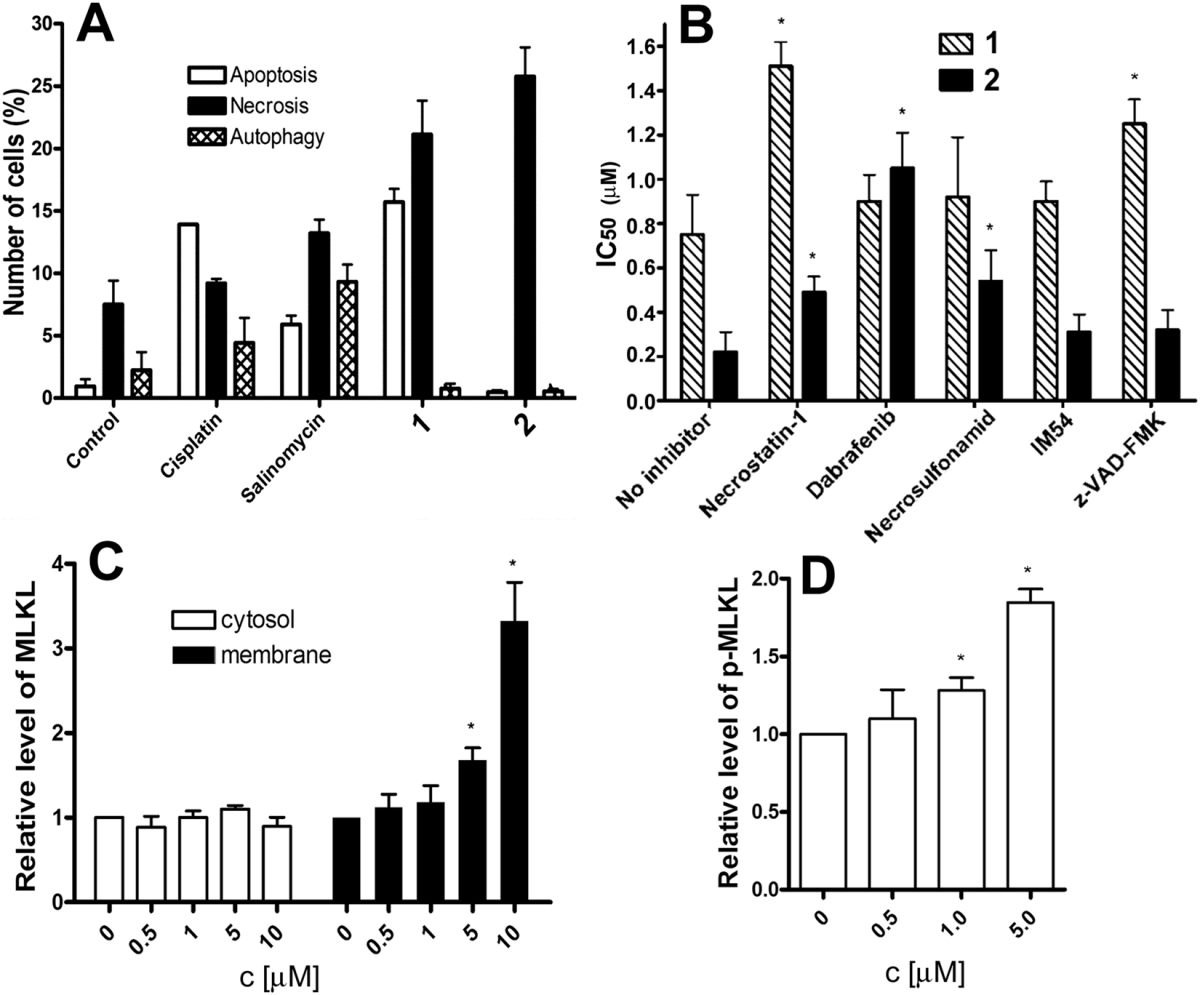
(**A**) Flow cytometric analysis of the mechanism of cell death after the treatment with the investigated compounds in MCF-7^CD44+/CD24−^ cells. Cells were treated with equitoxic concentrations of the tested compounds corresponding to their respective IC_50,72h_ values (Table 1). Modes of cell death were determined by staining with Annexin-V (apoptosis), propidium iodide (necrosis) and Cyto-ID (autophagy) with subsequent analysis by flow cytometry. (**B**) Graphical representation of the IC_50_ values of Ru(II) complex **1** and Os(II) complex **2** against MCF-7^CD44+/CD24−^ cells in the absence and presence of necrostatin-1 (20 μM), dabrafenib (10 μM), necrosulfonamid (2.5 μM), IM-54 (10 μM) and z-VAD-FMK (10 μM) after 72 h- co-incubation. Error bars represent standard deviations, n = 3. The symbol (*) denotes a difference (*p* < 0.05) from a sample incubated with the investigated compound in the absence of the inhibitor. (**C**) Relative levels of MLKL protein in the cytosol (white columns) and membrane (black columns) after the treatment of MCF-7^CD44+/CD24−^ cells with Os(II) complex **2** at the indicated concentration for 72 h. (**D**) Relative levels of phosphorylated MLKL protein (p-MLKL) after the treatment of MCF-7^CD44+/CD24−^ cells with **2** at indicated concentration for 72 h. In both panels C and D, the data represent a mean and standard deviation from three independent experiments. The symbol (*) denotes a difference (*p* < 0.05) from control, untreated cells.

Basic types and mechanisms of cell death are apoptosis, autophagy, or necrosis^26^. The levels of apoptosis, autophagy, and necrosis induced in MCF-7^CD44+/CD24−^ cells cultured as 3D spheroids treated with equitoxic concentrations of Ru(II) complex **1**, Os(II) complex **2**, cisplatin and salinomycin for 24 h were analyzed by flow cytometry. After the treatment, the cells were dissociated to the single cells and stained step by step with CYTO-ID^®^ autophagy detection probe, annexin-V as the apoptosis marker together with propidium iodide (PI) as a necrosis marker. Flow cytometric analysis of MCF-7^CD44+/CD24−^ cells showed (Figs 4A and S11) that Ru(II) complex **1** and salinomycin appear to kill MCF-7^CD44+/CD24−^ cells mainly by the necrosis mechanism, although apoptosis has also been implicated and in the case of salinomycin also a population of cells was classified as an autophagy fraction. Unsurprisingly, cisplatin appears to kill MCF-7^CD44+/CD24−^ cells mainly by the apoptosis.

Notably, the results shown in Figs 4A and S11 are consistent with the hypothesis that the necrosis mechanism of cell death may be the major mechanism of MCF-7^CD44+/CD24−^ cell death induced by Os(II) complex **2**. However, necrosis is the final stage that cells undergo long after dying, whereas apoptosis, oncosis, and necroptosis are mechanisms of programmed cell death^27–29^. Interestingly, it was shown recently^10^ that a mechanism of cell death induced by Os(II) complex **2** in triple-negative breast cancer cells MDA-MB-231 relies on oncosis. Thus, because Os(II) complex **2** did not kill MCF-7^CD44+/CD24−^ cells by apoptosis or autophagy mechanisms, it is reasonable to assume that the major mechanism of programmed death of MCF-7^CD44+/CD24−^ cells induced by Os(II) complex **2** is an oncosis or necroptosis, genetically controlled forms of programmed cell death. Notably, necroptosis has been induced in CSCs by a nickel(II) dithiocarbamate phenanthroline complex^30^.

To verify this hypothesis, we assessed first the effect of complexes **1** and **2** on the expression of the surface receptor porimin in the MCF-7^CD44+/CD24−^ cells. The protein porimin (pro-oncosis receptor inducing membrane injury) is a known marker of oncosis^31–33^. Porimin induces the aberrant membrane permeability responsible for the whole cell swelling during oncosis^32^. No significant increase in the expression of porimin as a consequence of the treatment of MCF-7^CD44+/CD24−^ cells with **1** or **2** was observed (Supplementary Fig. S12). This result suggests that oncosis is not a major mechanism of cell death induced by complexes **1** and **2**.

Another programmed form of necrosis is necroptosis. Necroptosis signaling requires the receptor interacting protein kinases 1 and 3 (RIP1 and RIP3), which together with the pseudokinase mixed lineage kinase domain-like (MLKL) form the necrosome^34^. Hence, MLKL is a downstream effector of necroptosis that becomes normally activated upon phosphorylation by RIP3^35^. Therefore, we next investigated, with the aid of antiproliferation and PI staining studies using flow cytometry, whether the major mechanism of MCF-7^CD44+/CD24−^ cell death induced by complexes **1** and **2** is associated with necroptosis. We co-incubated complex **1** or **2** with specific inhibitors of apoptosis, unregulated and necroptotic necrosis pathways and the viability of MCF-7^CD44+/CD24−^ cells was determined by the standard 3-(4,5-dimethylthiazol-2-yl)-2,5-diphenyltetrazolium bromide (MTT) test. IM-54 is a potent and selective inhibitor of unregulated necrosis induced by oxidative stress^36^, and co-incubation of **1** or **2** with this inhibitor did not significantly affect the potency of **1** and **2** to kill MCF-7^CD44+/CD24−^ cells (Fig. 4B). This result confirms that **1** and **2** do not kill MCF-7^CD44+/CD24−^ cells by the unregulated necrosis mechanism. Co-incubation of **1** with *z*-VAD-FMK, an irreversible and potent inhibitor of caspase-dependent apoptosis, markedly decreased the potency of **1** towards MCF-7^CD44+/CD24−^ cells (Fig. 4B) providing further evidence for the ability of complex **1** to kill MCF-7^CD44+/CD24−^ cells by an apoptotic mechanism. However, flow cytometric analysis of MCF-7^CD44+/CD24−^ cells showed (Fig 4A and Supplementary S11) that Ru(II) complex **1** effectively killed these cells also by the necrosis mechanism. Consistent with this observation is the finding that co-incubation of **1** with necrostatin-1, an inhibitor of the RIP1 kinase that prevents the necroptotic cell death^37^ significantly decreased the potency of **1** against MCF-7^CD44+/CD24−^ cells. Co-incubation of Os(II) complex **2** with three specific inhibitors of necroptosis, namely necrostatin-1, dabrafenib (an inhibitor of RIP3 kinase^38^) and necrosulfonamid (an inhibitor of necroptosis by blocking MLKL protein downstream of the activation of RIP3^39^), significantly decreased potency towards MCF-7^CD44+/CD24−^ cells (Fig. 4B). Also importantly, co-incubation of Os(II) complex **2** with *z*-VAD-FMK affected the potency of **2** towards MCF-7^CD44+/CD24−^ cells only negligibly (Fig. 4B) suggesting that **2** does not kill MCF-7^CD44+/CD24−^ cells by an apoptotic mechanism. Taken together, the antiproliferation data suggest that necroptosis seems to be a prominent mode of programmed death of MCF-7^CD44+/CD24−^ cells induced by Os(II) complex **2**. On the other hand, the mechanism of action of Ru(II) complex **1** in MCF-7^CD44+/CD24−^ cells involves blocking of both necroptosis and apoptosis.

Necroptosis is known to result in the release of the cellular components after plasma membrane disruption and permeabilization^34^. Thus, to further characterize the mode of cell death evoked by **1** and **2** and to verify that these complexes induce plasma-membrane permeabilization of MCF-7^CD44+/CD24−^ cells connected with necroptosis, propidium iodide (PI) staining studies were undertaken as well using flow cytometry. The cells which maintain their cell membrane integrity, such as those undergoing early-stage apoptosis, are not stained by PI^30^. In contrast, PI can accumulate in necrotic cells, and stain them due to their disrupted cell membranes^30^. MCF-7^CD44+/CD24−^ cells treated with **1** and **2** (1.5 or 0.5 µM, respectively, for 24 h) displayed increased PI uptake relative to untreated cells, suggesting necrotic cell death (Supplementary Fig. S13). In the presence of an inhibitor of unregulated necrosis IM-54, the increased PI uptake by MCF-7^CD44+/CD24−^ cells treated with **1** and **2** was unaffected. Co-incubation of **1** with *z*-VAD-FMK (10 µM) or necrostatin-1 (20 µM) decreased PI uptake only partially (Supplementary Fig. S13A) suggesting the involvement of both apoptotic and necrosis mechanisms in killing MCF-7^CD44+/CD24−^ cells by Ru(II) complex **1**. Notably, co-incubation of Os(II) complex **2** with *z*-VAD-FMK (10 µM) did not affect increased PI uptake relative to untreated cells, whereas co-incubation of **2** with three specific inhibitors of necroptosis, necrostatin-1, dabrafenib or necrosulfonamid, almost completely blocked PI uptake by MCF-7^CD44+/CD24−^ cells (Supplementary Fig. S135B). Thus, PI staining studies (Supplementary Fig. S13) are fully consistent with the antiproliferation studies (Fig. 4A) confirming that while mechanism of action of Ru(II) complex **1** in MCF-7^CD44+/CD24−^ cells involves blocking of both necroptosis and apoptosis, necroptosis seems to be a prominent mode of programmed death of MCF-7^CD44+/CD24−^ cells induced by Os(II) complex **2**.

To further explore this possibility, the immunoblotting analysis studies were performed with MCF-7^CD44+/CD24−^ cells. Necroptosis is associated with the formation of necrosomes which activate homo-oligomerization and phosphorylation of MLKL with the subsequent movement of the resulting MLKL homo-oligomers from the cytoplasm to the cellular membrane inducing membrane permeabilization^30,35,40^. Indeed, we saw (Supplementary Fig. S14) that MCF-7^CD44+/CD24−^ cells express necrosome MLKL marker. Notably, the level of expression of MLKL in cytosol remained unaltered upon treatment with Os(II) complex **2** (0.5–10 µM; Fig. 4C and Supplementary S14) whereas the quantity of the protein in the membrane fraction increased with the increasing concentration of Os(II) complex **2** indicating a translocation of MLKL to the membrane (Fig. 4C and Supplementary S14). Moreover, upon treatment of MCF-7^CD44+/CD24−^ cells with Os(II) complex **2** for 72 h (Fig. 4D and Supplementary S15), a concentration-dependent phosphorylation of MLKL was observed. Thus, the results of the immunoblotting experiments, demonstrating the effect of Os(II) complex **2** on phosphorylation of MLKL and its redisposition to the membrane, confirmed necroptosis as a prominent mode of death triggered in MCF-7^CD44+/CD24−^ cells by Os(II) complex **2**.

## Conclusions

In summary, we show that Os(II) complex **2** containing a dca ligand is capable of efficient killing CSCs in heterogeneous populations of human breast cancer cells MCF-7 and SKBR-3. Additionally, Os(II) complex **2** inhibits the formation of mammospheres by specifically targeting CD44-positive and CD24-negative CSC-like cells. Importantly, the potency of Os(II) complex **2** to kill CSCs in populations of MCF-7 and SKBR-3 is noticeably higher than that of salinomycin, one of the most selective CSC-targeting compounds hitherto identified and an analog of complex **2**, Ru(II) complex **1**. Moreover, Os(II) complex **2** has been shown to have little toxic effects in cultured non-cancerous mammary gland MCF-10A cells^10^ highlighting its selectivity for cancer cells. The antiproliferation and PI staining studies using flow cytometry studies suggest that Os(II) complex **2** induces hBCSC death predominantly by necroptosis, a programmed form of necrosis. On the other hand, mode of action of Ru(II) complex **1** and salinomycin in hBCSCs involves blocking of both necroptosis and apoptosis. The differences in the biological effects of Os(II) complex **2** and Ru(II) complex **1** or salinomycin might be connected with the ability of Os(II) complex **2** to transport into the cell biologically active dca in contrast to Ru(II) complex **1** and salinomycin. The lower potency of Ru(II) complex **1** in comparison with Os(II) complex **2** is apparently connected with a relatively quick release of the dca ligand due to the hydrolysis of Ru(II) complex before it enters the cells^10,41^. Dca has shown great potential as anticancer agent reverting the Wartburg effect as a specific “metabolic sign” reflecting the stem origin of the neoplastic cell^42^. Hence, it is reasonable to suggest that dca was able to contribute to the overall biological effects of Os(II) complex **2**.

Recently, the effect of another osmium complex, Os(VI) nitrido complex on bCSCs was reported^8^. The structure, chemical properties (oxidation number of osmium atom) and mechanism of action of the latter complex are, however, fundamentally different from those of the Os(II) complex **2** investigated in this study. Also importantly, the Os(II) complex **2** displayed significantly higher potency to reduce the number of viable cells in the mammospheres from CSC-enriched breast cancer cells in comparison with salinomycin, one of the most selective CSC-targeting compounds identified to date; in contrast, the potency of the Os(VI) nitrido complex was in comparison with salinomycin lower. Thus, we propose that the Os(II) bathophenanthroline complex investigated in this study belongs to an interesting class of human breast cancer stem cell-selective, mammosphere potent agents.

Experiments aimed at revealing further details of the unique effects of Os(II) complex **2** including the role of the dca ligand on hBCSCs are currently in progress. Collectively, the current study provides a solid research foundation for further preclinical and clinical studies and applications of Os(II) complex **2** to comply with the emergent need for bCSC-specific chemotherapeutics to overcome cancer relapse and metastases formation in the clinic.

## Methods

### Starting materials

Salinomycin, cisplatin, human serum albumin (HSA) and hEGF were from Sigma Aldrich (Darmstadt, Germany), B27 supplement, Dulbecco’s phosphate buffered saline (DPBS) and Corning CellStripper Dissociation Reagent (CSDR) from Thermo Fisher Scientific Inc. (MA, USA) and anti-CD44, anti-CD24 antibodies and isotype IgG controls from Miltenyi Biotec (Gladbach, Germany). Complexes [Ru(*η*^6^-*p*cym)(bphen)(dca)]PF_6_ (complex **1**) and [Os(*η*^6^-*p*cym)(bphen)(dca)]PF_6_ (complex **2**) ([*p*cym = 1-methyl-4-(propan-2-yl)benzene (*p*-cymene); bphen = 4,7-diphenyl-1,10-phenanthroline (bathophenanthroline); dca = dichloroacetate] were prepared and characterized as described previously^9^. MCF-7 cells were kindly supplied by Professor B. Keppler, University of Vienna (Austria) and SKBR-3 cells were from the European collection of cell cultures (ECACC) (Salisbury, UK). Aldefluor kit, diethylaminobenzaldehyde (*DEAB*) reagent, and *ACCUTASE™* were purchased from (StemCell^TM^ technologies, CA, USA). CYTO-ID^®^ autophagy detection kit was from Enzo Life Sciences (Farmingdale, New York, USA).

### Isolation of cancer stem cells

The human breast cancer cells MCF-7 and SKBR-3 were used as the primary culture for sorting of CSCs. The adherent cells were transferred as single cells to ultra-low attachment plastics (ULA; Corning, NY, USA), cultivated in DMEM medium supplemented with 2% B27 (2%), hEGF (20 ngmL^−1^) and HSA (0.15% (w/v)). These conditions led to the enrichment of the CSC population (CD44+/CD24−) in the MCF-7 and SKBR-3 cell cultures. Cells were incubated in serum-free conditions for 96 h and then subjected to fluorescently-activated cell sorting (FACS). The population of CD44+/CD24− cells was sorted for further experiments. The proportion of CD44+/CD24− was checked during the experiments.

### Formation of tumorspheres from MCF-7^CD44+/CD24−^ and SKBR-3^CD44+/CD24−^ cells

MCF-7^CD44+/CD24−^ and SKBR-3^CD44+/CD24−^ cells cultured in DMEM-F12 HAM medium supplemented with B27 supplement (2%) and hEGF (20 ngmL^−1^) were seeded in 96-well ultralow attachment plates (Corning) for 5 days. Mammospheres were counted, photographed by using Canon EOS 1200D camera attached to Olympus CKX41 inverted microscope with 10X/0.25 phase contrast objective and processed for further mammosphere size analysis by QuickPHOTO MICRO 3.1 program (PROMICRA, Prague, Czech Republic). Cell clumps were not considered.

### Viability assay of the mammospheres

The viability of the mammospheres was determined by CellTiter-Glo^®^ 3D cell viability assay. The MCF-7, SKBR-3, MCF-7^CD44+/CD24−^ and SKBR-3^CD44+/CD24−^ cells were transferred to 96-well ultra-low attachment plates (ULA; Corning, NY, USA), cultivated in DMEM medium supplemented with B27 (2%), epidermal growth factor (20 ngmL^−1^) and HSA (0.15% (w/v)) and cultivated for 144 h to form mammospheres. The mammospheres were subsequently treated with increasing concentrations of the investigated compounds and incubated for another 72 h. After the treatment period, the wells were filled with an equal amount of CellTiter-Glo^®^ 3D Reagent (100 μL), and the plates were vigorously mixed for 5 min followed by 25 min of incubation at room temperature. The viability of mammospheres was evaluated by measuring the luminescence using a fluorescence/luminescence reader Infinite200 (Tecan). The IC_50_ values were interpolated from the resulting curves constructed by plotting cell survival (%) versus drug concentration. The reported IC_50_ values are the average of three independent experiments, each consisting of three replicates per concentration level. The reading values were converted to the percentage of control (% cell survival).

### Inhibition of the formation of mammospheres from the suspension of MCF-7^CD44+/CD24−^ single cells

MCF-7 cells were used as the primary source of CSCs before FACS sorting. Cells were cultured under 3D forming conditions as described in the paragraph “Formation of tumorspheres from MCF-7^CD44+/CD24−^ and SKBR-3^CD44+/CD24−^ cells”. Then, the spheroids were dissociated to the single cells by using Accutase^TM^ (StemCell^TM^ Technologies, Canada) and sorted as the unique population of CD44+/CD24− cells (for further details see the section: Isolation of cancer stem cells). The investigated compounds were added to the suspension of MCF-7^CD44+/CD24−^ single cells and the cells were incubated for additional 5 days and analyzed using tumor formation assay.

### Monitoring the changes in the diameter of the preformed MCF-7^CD44+/CD24−^ spheroids

Sorted single cells prepared as described in the preceding paragraph were transferred to 96w ULA plates and cultured for 96 h. The spheroids with the mean diameter of about 200–250 μm were treated for 24–72 h at 37 °C with the investigated compounds. The analysis of the morphology of spheroids, visual scoring and determination of their diameter were performed every 24 h after the treatment commenced until 72 h. Samples were photographed by using the Canon EOS 1200D camera attached to Olympus CKX41 inverted microscope with 10X/0.25 phase contrast objective. Digital images were acquired and analyzed by QuickPHOTO MICRO 3.1 program (PROMICRA, Prague, Czech Republic).

### The effect on the heterogeneity of breast cancer cells

#### Monitoring CD^44^+/CD^24^− population in MCF-7 cells

To determine the effect of the investigated compounds on the heterogeneity of breast cancer cells, flow cytometric studies were carried out with the aim to reveal the changes in the proportion of CSC-like, CD44+/CD24− cells within a heterogeneous population of MCF-7 cells (unsorted, uninfluenced by the CSC-enrichment after FACS sorting). The adherent MCF-7 cells were transferred to ultra-low attachment plastics (ULA; Corning, NY, USA), cultivated in DMEM medium supplemented with B27 (2%), EGF (20 ngmL^−1^) and HSA (0.15% (w/v)). Cells were cultured for 96 h to form the spheroids and then treated for another 24 h with the investigated compounds at the concentrations corresponding to their respective values of IC_50,72h_. The cells were subsequently washed with DPBS and incubated with CSDR to prepare single cells from the preformed spheroids and processed for the staining with anti-CD44-APC and anti-CD24-PE antibodies (Miltenyi Biotec, Bergisch Gladbach, Germany) according to the manufacturer instructions. The heterogeneity of surface markers on MCF-7 cells was determined by using flow cytometry (BD FACSVerse, BD Biosciences, San Jose, CA, USA). The data were analyzed using FCS Express 6 software (DeNovo software; Glendale, CA, USA).

#### Monitoring CD^44^+/CD^24^− population and ALDH activity in SKBR-3^CD44^+/^CD24^− cells

The effects of the investigated compounds on the heterogeneity of breast cancer cells were also investigated in ALDH positive CSC-enriched SKBR-3^CD44+/CD24−^ cells. The use of this cell line makes it possible to analyze by flow cytometry fluorescence properties of the indicator of ALDH activity [BODIPY-aminoacetate (BAA)] along with that of the antibodies for CD44/CD24 cell surface markers stained with APC or PE.

The adherent CSC-enriched SKBR-3^CD44+/CD24−^ cells were prepared as described in the paragraph Isolation of cancer stem cells (*vide supra*). After FACS sorting, cells were incubated under the ultra-low attachment conditions for 96 h and subsequently treated with the investigated compounds at the equitoxic concentrations corresponding to their respective IC_50,72h_ values for another 24 h and further analyzed for ALDH activity and CSC markers. Then, the cells were washed in DPBS and incubated in CSDR to disrupt spheroids and cell clumps. After this step, the cells were washed again in DPBS and incubated in the Aldefluor Assay buffer for 40 min at 37 °C. Negative controls supplemented with DEAB reagent (a commonly used inhibitor of ALDH enzymes in CSC biology) were analyzed in parallel. The samples were centrifuged and incubated with anti-CD44-APC and anti-CD24-PE antibodies. Isotype controls mouse IgG1 (APC/PE) were used at the same concentrations as the primary antibodies. Cells were controlled for their viability during the flow cytometric analysis with DAPI dye, and only viable cells were analyzed for their CD44/CD24/ALDH profile. The data (2 × 10^4^ events) were analyzed using FCS Express 6 (DeNovo software; Glendale, CA, USA). The dot plots are the representatives of three independent experiments. Negative ALDH cells (gating for ALDH positivity) were tested after the treatment of the corresponding sample with the DEAB reagent, which provides control for setting the gates in this flow-cytometric analysis.

### Determination of mode of cell death

Apoptotic, necrotic and autophagic mechanisms of cell death were measured using the flow cytometry. MCF-7^CD44+/CD24−^ cells were cultured as 3D spheroids for 96 h. The spheroids were treated with the investigated compounds at the concentrations corresponding to their respective IC_50,72h_ values determined for MCF-7^CD44+/CD24−^ by using Cell Titer-Glo (31 μM cisplatin, 7 μM salinomycin, 1.3 μM **1** and 0.6 μM **2**) and incubated at 37 °C in a humidified 5% CO_2_ atmosphere for 24 h. The spheroids were dissociated to the single cells by using Accutase^TM^ cell detachment solution, pelleted, stained with CYTO-ID^®^ autophagy detection kit and incubated for another 30 min in the culture medium in the humidified CO_2_ incubator. The cells were subsequently centrifuged for 3 min at 300 g and stained with PI (1 μgmL^−1^) and annexin-V pacific blue conjugate (5 μL per 100 μL of the cell suspension) for 15 min at room temperature. Cells were analyzed using the flow cytometer (BD FACSVerse).

The viability of MCF-7^CD44+/CD24−^ cells treated with **1** or **2** in the presence of specific inhibitors of apoptosis, unregulated and necroptotic necrosis pathways was assayed by colorimetric MTT assay. Cells were seeded on 96w plates at the density of 4000 cells/well. After overnight incubation, the cells were treated with the increasing concentration of **1** or **2** in the presence of the specific inhibitors: necrostatin-1, dabrafenib, necrosulfonamid, IM-54, and z-VAD-FMK. After 72 h of incubation, 20 μL of MTT solution (1.25 mgmL^−1^) was added to each well, and plates were incubated for 4 h. At the end of the incubation time, the medium was removed, and the formazan product was dissolved in 100 μL of DMSO per well. Cell viability was evaluated by measurement of the absorbance at 570 nm (reference wavelength at 620 nm) using an Absorbance Reader Sunrise Tecan Schoeller. IC_50_ values were calculated from curves constructed by plotting cell survival (%) versus drug concentration (μM). All experiments were done in triplicate. The final concentrations of tested compounds in cell culture medium were verified by FAAS.

### Propidium iodide (PI) uptake

Untreated and treated MCF-7^CD44+/CD24−^ cells (2 × 10^5^ cells/well) grown in six-well plates were washed with PBS (1 mLx3), harvested, incubated with PI (3.3 µgmL^−1^ for 20 min), and analyzed by using flow cytometer (BD FACSVerse, BD Biosciences, San Jose, USA). Cell populations were analyzed using FCS Express 6 software (DeNovo software; Glendale, CA).

### Immunoblotting experiments

MCF-7^CD44+/CD24−^ were seeded at a density of 10^6^ cells/dish, grown overnight and incubated with Os(II) complex **2** (0.5, 1, 5, and 10 µM) for 72 h. Following washing with PBS, the cells were scraped into PBS and pelleted. Cytosolic and membrane fractions were separated using FractionPREP^TM^ Cell Fractionation kit (BioVision) following the manufacturer’s protocol. Individual fractions were immediately combined with an identical volume of 2x Laemmli loading buffer (125 mM Tris-HCl, 20% glycerol, 10% 2-mercaptoethanol, 4% SDS and 0.004% bromophenol blue) and heated at 95 °C for 10 min. The fractions were resolved by 4–15% SDS-PAGE (100 V, 60 min), followed by electro-transfer to PVDF membrane (20 V, 1 h). Membranes were blocked in 4% non-fat milk in PBST (0.1% Tween 20 in PBS) and incubated with primary antibodies (anti-MLKL and anti-MLKL[phosphor S358], Abcam) overnight (4 °C). Following the incubation with horseradish peroxidase-conjugated secondary antibodies (Abcam), the immune complexes were detected using SignalFire^TM^ ECL Reagent (Cell Signaling Technology) with chemiluminescence imager Amersham 680.

### Other physical methods

The platinum, ruthenium and osmium contents were quantified by FAAS with a Varian AA240Z Zeeman atomic absorption spectrometer equipped with a GTA 120 graphite tube atomizer or ICP-MS with an Agilent 7500 spectrometer (Agilent, Japan).

### Statistical analysis

Statistical analysis of experimental data was carried out using an analysis of variance (ANOVA) and non-parametric student´s t-test. The symbols (**) and (*) denote a significant difference (p < 0.001 and p < 0.05), respectively, from the untreated control.

## Supplementary information

Supporting information

## Data Availability

All data and results have been added to this manuscript and the Supplementary Material Section.
