# Supplementary material for: An anticancer Os(II) bathophenanthroline complex as a human breast cancer stem cell-selective, mammosphere potent agent that kills cells by necroptosis
Source: Sci Rep. 2019 Sep 16;9:13327. doi: 10.1038/s41598-019-49774-x (PMC6746710; doi:10.1038/s41598-019-49774-x)
Supplement: Supplementary file 1 — Supporting information [file 41598_2019_49774_MOESM1_ESM.pdf]

Electronic Supplementary Information

An anticancer Os(II) bathophenanthroline complex as a human breast cancer stem cell-selective, mammosphere potent agent that kills cells by necroptosis

Vojtech Novohradsky[1], Lenka Markova[1], Hana Kostrhunova[1], Zdeněk Trávníček[2], Viktor Brabec[1,3], Jana Kasparkova[a]*

[1]Czech Academy of Sciences, Institute of Biophysics, Kralovopolska 135, 612 65 Brno, Czech Republic. *e-mail: jana@ibp.cz.*
[2]Division of Biologically Active Complexes and Molecular Magnets, Regional Centre of Advanced Technologies and Materials, Šlechtitelů 27, 783 71 Olomouc, Czech Republic
[3]Department of Biophysics, Faculty of Science, Palacky University, 17. listopadu 12, CZ-77146 Olomouc, Czech Republic

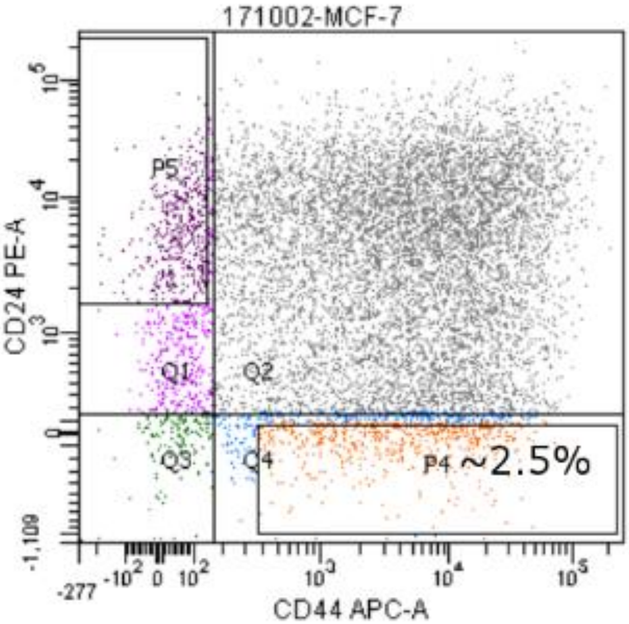

Supplementary Fig. S1: FACS with CD24 and CD44 markers demonstrating the proportion of the MCF7 cells carrying the CD44+/CD24- profile associated with CSCs. The percentage of CD44+/CD24- cells (gate-P4) was approximately 2.5%. X and Y axes: Fluorescence intensity of APC-labeled anti-CD44 antibody and fluorescence intensity of PE-labeled anti-CD24 antibody, respectively.

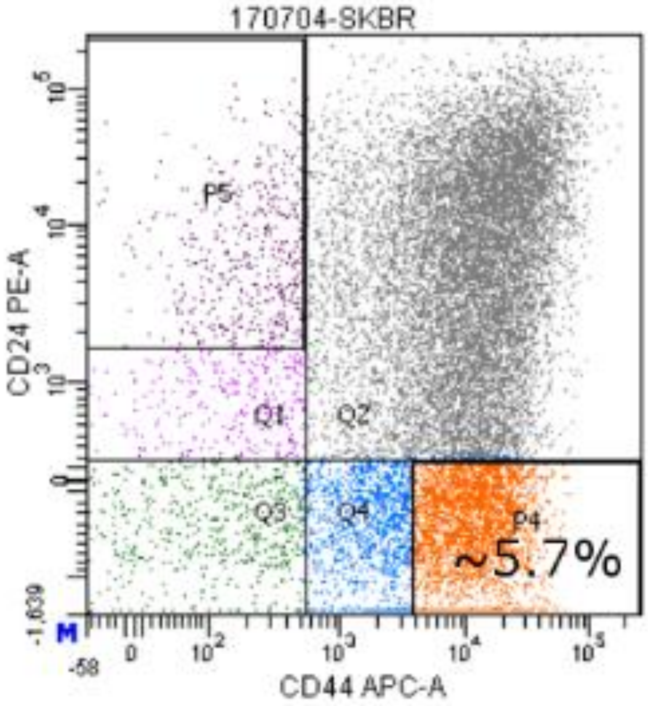

Supplementary Fig. S2: FACS with CD24 and CD44 markers demonstrating the proportion of the SKBR-3 cells carrying the CD44+/CD24- profile associated with CSCs. The percentage of CD44+/CD24- cells (gate-P4) was approximately 5.7%. X and Y axes: Fluorescence intensity of APC-labeled anti-CD44 antibody and fluorescence intensity of PE-labeled anti-CD24 antibody, respectively.

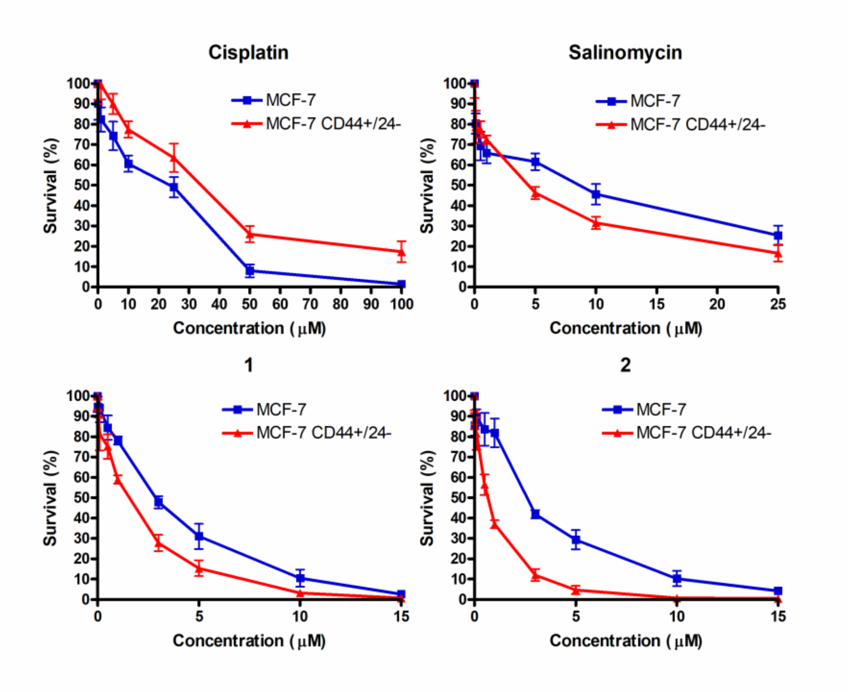

Supplementary Fig. S3: Representative dose-response curves for the treatment of MCF-7 and MCF-7$^{CD44+/CD24-}$ cells with cisplatin(top left panel), salinomycin (top right panel), Ru(II) complex **1** (bottom left panel), and Os(II) complex **2** (bottom right panel) after 72 h incubation. Error bars are the SDs.

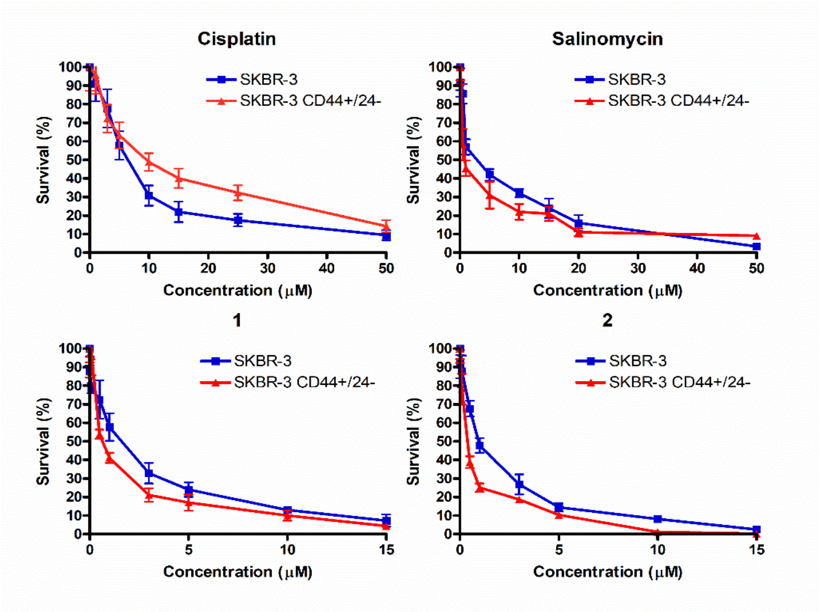

Supplementary Fig. S4: Representative dose-response curves for the treatment of SKBR-3 and SKBR-3$^{CD44+/CD24-}$ cells with cisplatin (top left panel), salinomycin (top right panel), Ru(II) complex **1** (bottom left panel), and Os(II) complex **2** (bottom right panel) after 72 h incubation. Error bars are the SDs.

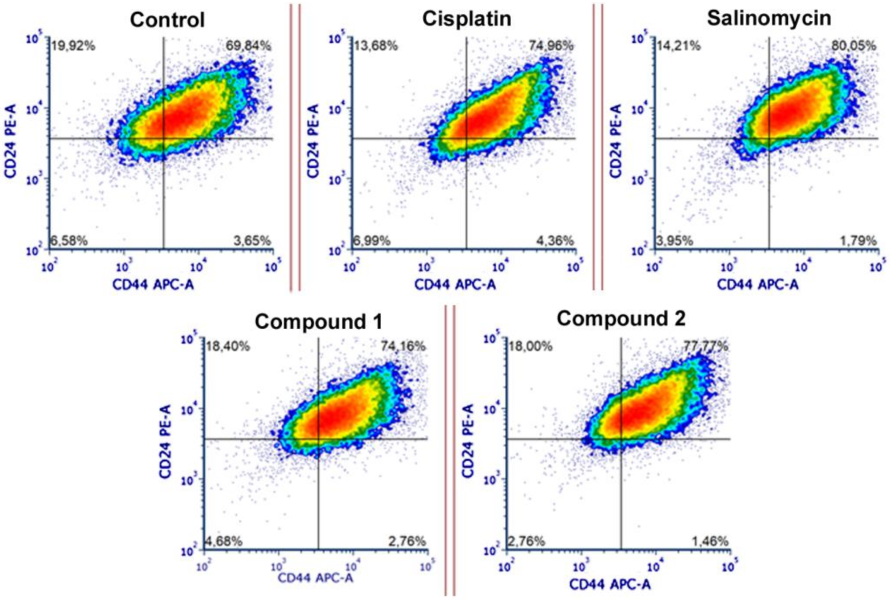

Supplementary Fig. S5: Representative flow cytometric analysis displaying the effect of the investigated compounds on the proportion of the cells carrying the CD44+/CD24- profile associated with CSCs within a heterogeneous population of MCF-7 cells. Cells were grown as 3D spheroids and treated for 24 h with the investigated compounds at the concentrations corresponding to their respective $IC_{50,72h}$ values. X and Y axes: Fluorescence intensity of APC-labeled anti-CD44 antibody and fluorescence intensity of PE-labeled anti-CD24 antibody, respectively.

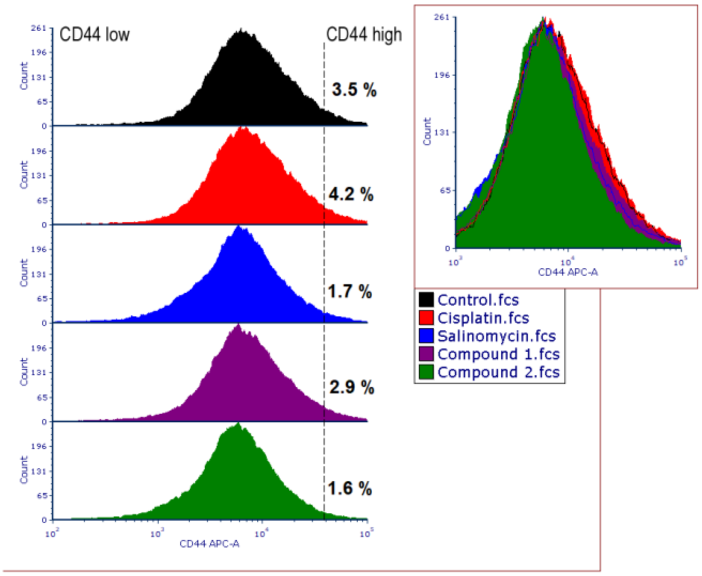

Supplementary Fig. S6: Representative histograms displaying the fluorescence emitted by anti-CD44-APC antibody stained MCF-7 cells cultured under 3D spheroid-forming conditions. Cells were treated for 24 h with the investigated compounds at the concentrations corresponding to their respective $IC_{50,72h}$ values. X axis: Fluorescence intensity of APC-labeled anti-CD44 antibody.

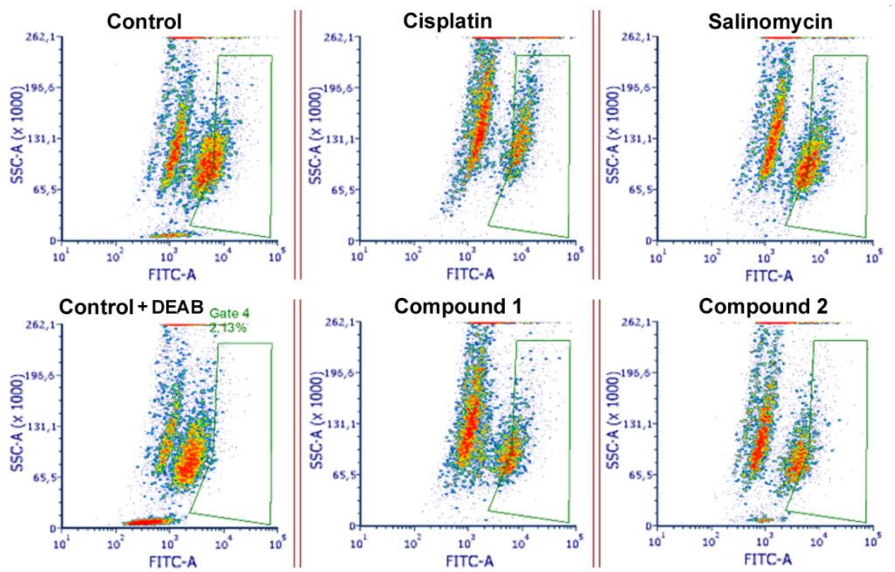

Supplementary Fig. S7: Representative flow cytometric analysis of the ALDH-positive populations within SKBR-3$^{CD44+/CD24-}$ cells. Cells were treated for 24 h with the investigated compounds at the concentrations corresponding to their respective $IC_{50,72h}$ values. Cells treated with the DEAB reagent (a potent inhibitor of cytosolic ALDH enzymes) were taken as a negative control. X and Y axes: Fluorescence intensity of Aldefluor and fluorescence intensity of SSC (side-scatter), respectively.

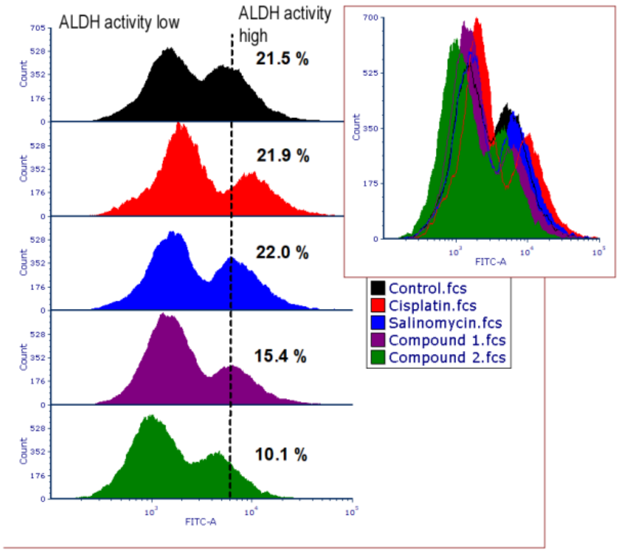

Supplementary Fig. S8: Representative histograms displaying the fluorescence emitted by SKBR-3$^{CD44+/CD24-}$ cells cultured in 3D spheroid-forming conditions stained with aldefluor reagent. Cells were treated for 24 h with the investigated compounds at the concentrations corresponding to their respective $IC_{50,72h}$ values. X axis: Fluorescence intensity of Aldefluor.

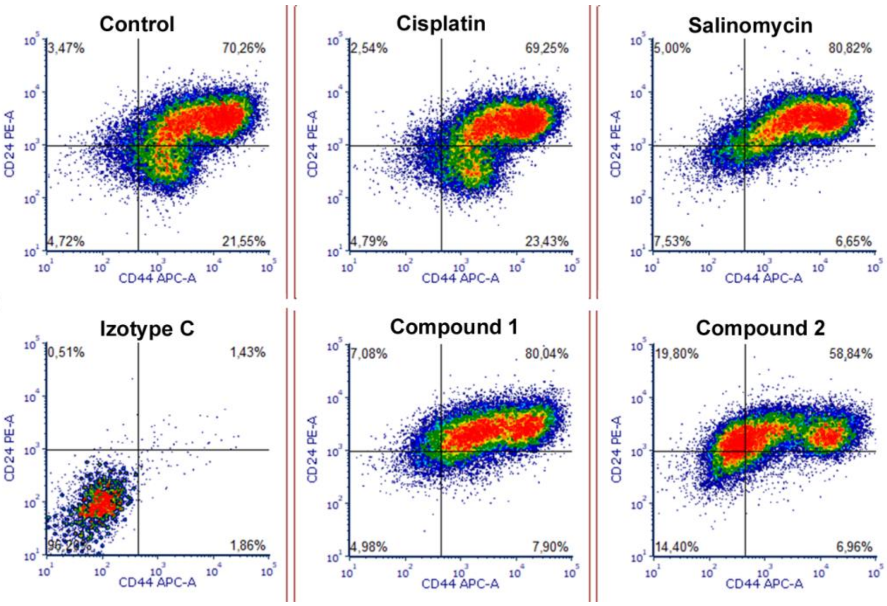

Supplementary Fig. S9: Representative flow cytometric analysis displaying the inhibitory effects of the investigated compounds on SKBR-3$^{CD44+/CD24-}$ cells. The sorted cells were treated for 24 h with the investigated compounds at the concentrations corresponding to their respective $IC_{50,72h}$ values and assayed for the expression of the CD44/CD24 cell surface markers. X and Y axes: Fluorescence intensity of anti-CD44 antibody conjugated with APC and fluorescence intensity of anti-CD24 antibody conjugated with PE, respectively.

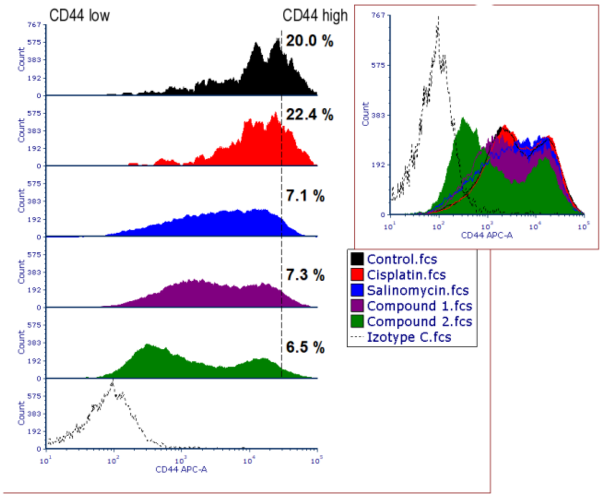

Supplementary Fig. S10: Representative histograms displaying the fluorescence emitted by anti-CD44-APC antibody stained SKBR-3$^{CD44+/CD24-}$ cells cultured under 3D spheroid-forming conditions. Cells were treated for 24 h with the investigated compounds at the concentrations corresponding to their respective $IC_{50,72h}$ values. X axis: Fluorescence intensity of Aldefluor.

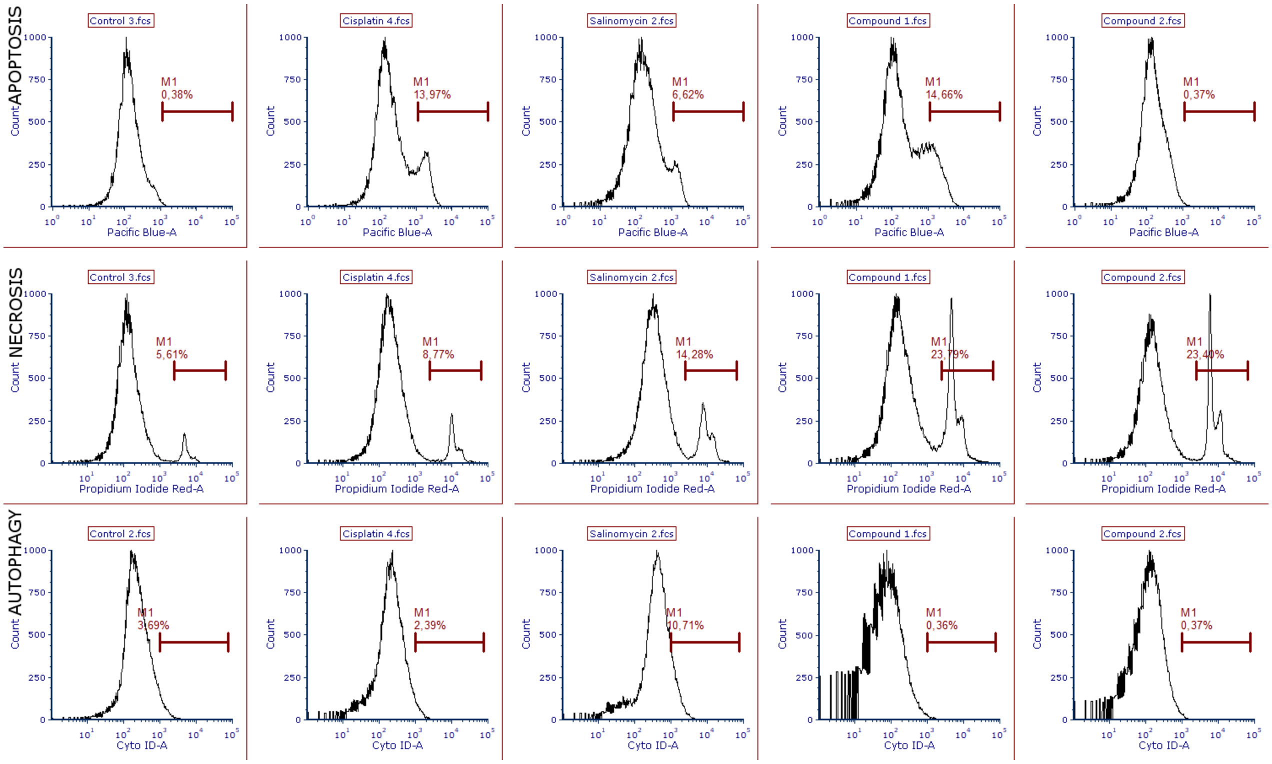

Supplementary Fig. S11: Flow cytometric analysis of the mechanism of the cell death after the treatment with the investigated compounds in MCF-7$^{CD44+/CD24-}$ cells.

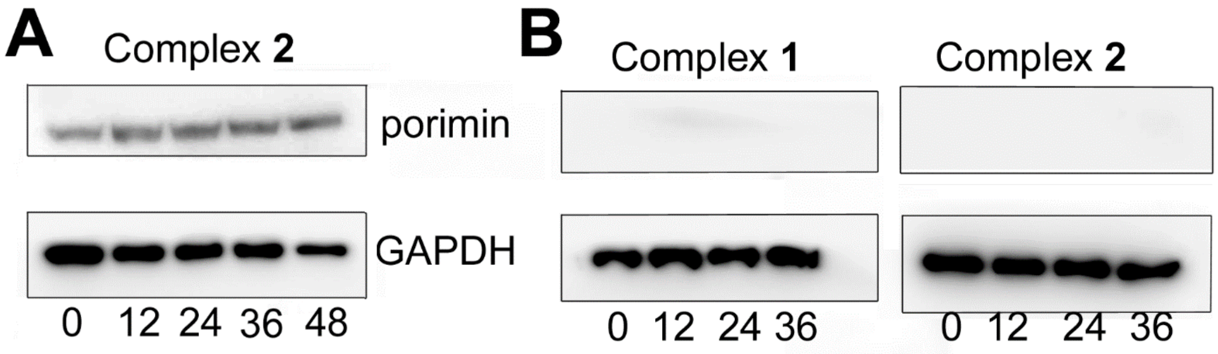

Supplementary Fig. S12: Representative western blotting demonstrating the effect of Ru(II) complex **1** and Os(II) complex **2** on porimin expression in MDA-MB-231 cells (A) (similar as in ref.[1]) or MCF-7$^{CD44+/CD24-}$ cells (B). The cells were treated with 1 μM metal complex for the time indicated in the figure. Twenty and 50 μg of total protein were loaded into the gels shown in both panels A and B.

1. Pracharova J., *et al.* Half-sandwich Os(II) and Ru(II) bathophenanthroline complexes: anticancer drug candidates with unusual potency and cellular activity profile in highly invasive triple-negative breast cancer cells. *Dalton Trans.* **47**, 12197 - 12208 (2018).

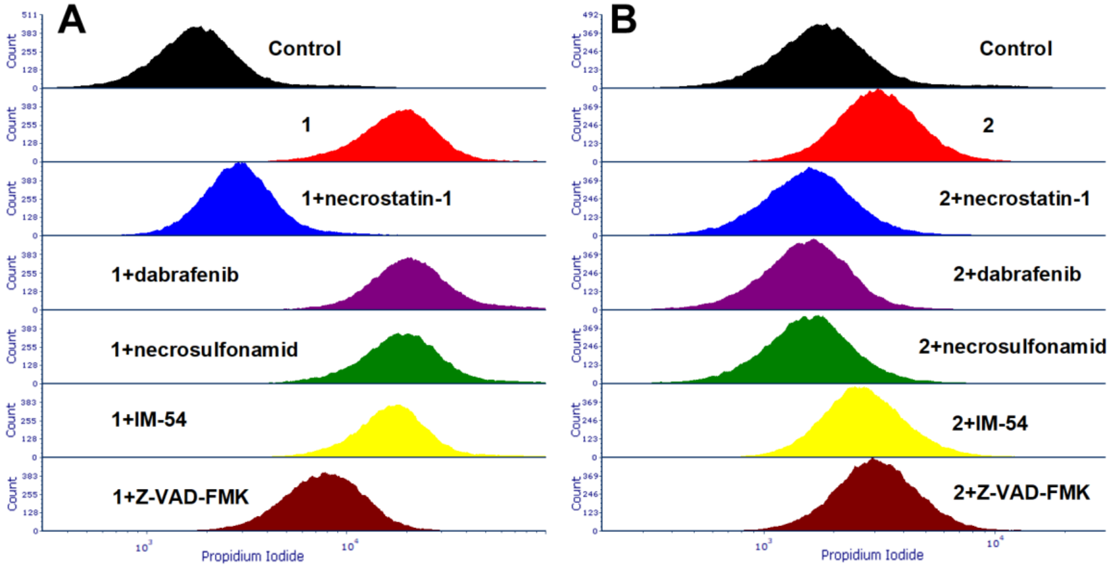

Supplementary Fig. S13: A. Representative histograms displaying the red fluorescence emitted by PI stained MCF-7$^{CD44+/CD24-}$ cells untreated (control), treated with Ru(II) complex **1** (1.5 μM for 24 h) or co-incubated for 24 h with Ru(II) complex **1** (1.5 μM) plus necrostatin-1 (20 μM), dabrafenib (10 μM), necrosulfonamid (2.5 μM), IM-54 (10 μM) and z-VAD-FMK (10 μM). Fifty thousands single cells were analyzed and plotted on the bi-exponential scale. B. Representative histograms displaying the red fluorescence emitted by PI stained MCF-7$^{CD44+/CD24-}$ cells untreated (control), treated with **2** (0.5 μM for 24 h) or co-incubated for 24 h with **2** (1.5 μM) plus necrostatin-1 (20 μM), dabrafenib (10 μM), necrosulfonamid (2.5 μM), IM-54 (10 μM) and z-

VAD-FMK (10 μM). Fifty thousands single cells were analyzed and plotted on the bi-exponential scale.

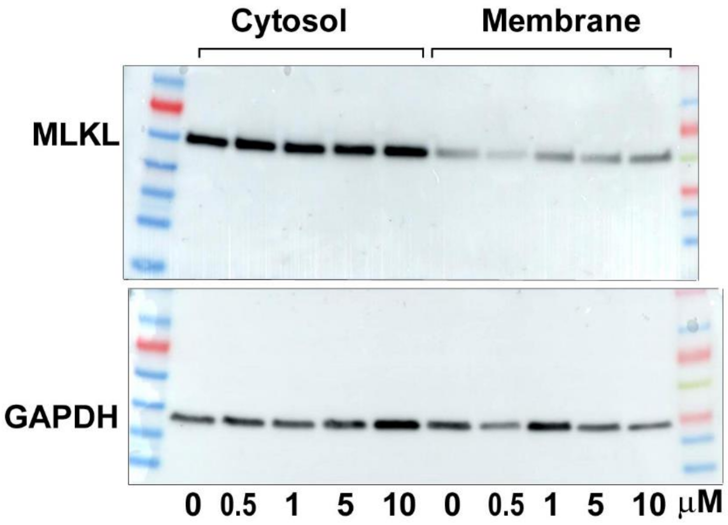

Supplementary Fig. S14: Immunoblotting analysis of MLKL expression in the cytosol and membrane fractions of MCF-7$^{CD44+/CD24-}$ cells treated with Os(II) complex **2** at the indicated concentrations for 72h. Cytosol and membrane fractions were resolved by SDS PAGE and analyzed by immunoblotting against MLKL. GAPDH was used as a loading control.

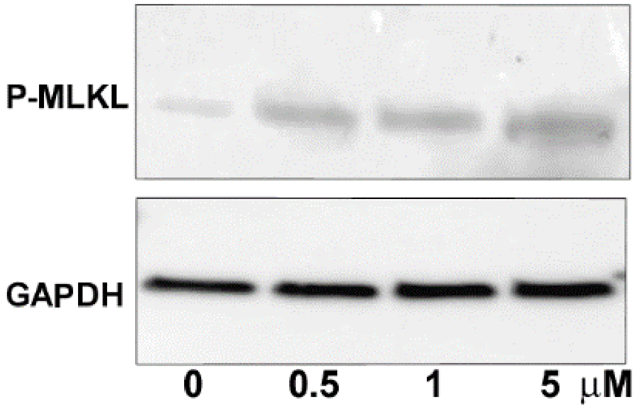

Supplementary Fig. S15: Immunoblotting analysis of phosphorylated MLKL (P-MLKL) in MCF-7$^{CD44+/CD24-}$ cells treated with Os(II) complex **2** (0.5, 1, and 5μM) for 72 h. Whole cell lysates were resolved by SDS-PAGE and analyzed by immunoblotting against phosphorylated MLKL and GAPDH (loading control).
